# Clinical outcomes and their relationship with tigecycline exposure in critically ill patients with drug resistant infections

**DOI:** 10.3389/fcimb.2026.1868760

**Published:** 2026-09-16

**Authors:** Wei Su, Shuping Song, Qing Tang, Binbin Feng, Yinshan Wu, Haitao Yu, Feng Guo, Zhenwei Yu

**Affiliations:** 1 Intensive Care Unit, Sir Run Run Shaw Hospital, School of Medicine, Zhejiang University, Hangzhou, China; 2 Research Center for Clinical Pharmacy, Zhejiang University, Hangzhou, China; 3 Department of Pharmacy, Sir Run Run Shaw Hospital, School of Medicine, Zhejiang University, Hangzhou, China; 4 Clinical Lab, Sir Run Run Shaw Hospital, School of Medicine, Zhejiang University, Hangzhou, China

**Keywords:** AUC, carbapenem-resistant, clinical response, mortality, tigecycline

## Abstract

**Background:**

Dosing tigecycline, an important last resort for carbapenem-resistant organisms, should be under the guidance of pharmacokinetic/pharmacodynamic (PK/PD) theory. However, tigecycline has shown large interindividual variability in PK among different patients, and its PK/PD target has limited data support. This study aimed to evaluate the clinical outcome and the relationship with exposure in critically ill patients with drug-resistant infections.

**Methods:**

A single center, prospective observational, single-arm cohort study was performed to assess the outcome and its relationship with exposure in critically ill patients with drug-resistant pathogen infections. Patients were enrolled according to the inclusion and exclusion criteria. The clinical outcomes, including clinical response rate, 28-day mortality rate, and major adverse events (AEs), were followed up. Plasma concentrations in steady status were intensively collected and determined for tigecycline concentration, and the 24-hour area-under-curves (AUCs) were calculated. The exposure-clinical outcome relationship was analyzed, and multivariate logistic regressions were performed to identify risk factors that associated with outcome.

**Results:**

A total of 130 patients were included. The most common infection type was low respiratory tract infection (76/130), and carbapenem-resistant *Acinetobacter baumannii* was the most common pathogen (64/130). The median tigecycline treatment length was 12 days. The median AUC was 14.0 mg h/L. The overall response rate and 28-day mortality rate were 50.0% and 36.2%, respectively. A lower fibrinogen level was the most common AE (51/130). ALB, PLT and combined use of cefoperazone-sulbactam were independent factors associated with clinical response, but AUC was negatively associated with clinical response.

**Conclusion:**

The clinical outcome of tigecycline in critically ill patients with resistant pathogen infection was satisfactory, but negative relationship between tigecycline exposure and outcome was found and the PK/PD profile in these patients should be revisited.

## 1 Introduction

Resistance in pathogenic microbes is increasing and becoming a global crisis due to the overuse and misuse of antibiotics (Aggarwal et al., 2024). The emergence and spread of carbapenem-resistant pathogens, which are resistant to most antibiotics, have caused high mortality and an enormous economic burden (Jean et al., 2022). According to CHINET surveillance data, the prevalence of carbapenem-resistant *Klebsiella pneumoniae* (CRKP) showed a remarkable increasing trend from 2.9% (imipenem resistance) in 2005 to 25.0% in 2018, and then slightly decreased to 22.6% in 2022 (Qin et al., 2024). The mortality of CRKP infection ranged from 12-28% among different religions (Wang et al., 2022). Tigecycline is a broad spectrum glycylcycline class antibiotic and remains susceptible to major clinically important drug-resistant pathogens, including carbapenem-resistant *Acinetobacter baumannii* (CRAB), CRKP, and methicillin-resistant *Staphylococcus aureus* (MRSA) (Wu et al., 2024). It is eliminated mainly through nonrenal routes, its dose does not need to be adjusted according to renal function, and its tissue penetration is high. Due to its easy dosing and activity against drug-resistant pathogens, tigecycline is widely used and plays an important role in the treatment of nosocomial infections (Yaghoubi et al., 2022).

However, some reports have suggested that an increase in the all-cause mortality rate is correlated with tigecycline therapy, especially for the population with hospital-acquired pneumonia (HAP) and ventilator-associated pneumonia (VAP) (McGovern et al., 2013). Experts attribute this finding to suboptimal tigecycline exposure at the targeted site (Leng et al., 2021). Dosing antibiotics should be under the guidance of pharmacokinetic/pharmacodynamic (PK/PD) theory. This may be more important for ICU patients, as they always have more severe infections or less susceptive pathogens and suboptimal exposure may lead to unfavorite outcomes (Dyer et al., 2024). Tigecycline has shown large interindividual variability in PK among different patients; moreover, its current PK/PD target, AUC/MIC ranged from 6.96 to 17.9 for different patients settings, has limited evidence support and is inconclusive (Leng et al., 2021; Dai et al., 2025). Prospective data linking tigecycline exposure (e.g., AUC) to clinical outcomes in critically ill patients with documented drug-resistant infections are limited, and the optimal exposure range is unclear. Therefore, we performed this prospective observational single-arm cohort study to assess the clinical outcomes and the relationship with tigecycline exposure in ICU patients with drug-resistant pathogen infections.

## 2 Methods

### 2.1 Study design

This study was designed as a single-center prospective observational single-arm cohort study. Ethics approval was obtained from the Ethics Committee of Sir Run Run Shaw Hospital, Zhejiang University School of Medicine (reference number KEYAN20190108-9). Written informed consent was obtained from the patient or patient representatives. The study duration was from May 2019 to December 2022.

### 2.2 Patient inclusion

The investigator searched for patients who met the following inclusion criteria consecutively: (1) hospitalized in the intensive care unit (ICU); (2) empirical or targeted treatment with tigecycline was expected due to suspected or confirmed drug-resistant pathogen infection, which were defined as resistant to carbapenem or at least two classes of antibiotics; and (3) patient or patient representative volunteered to participate and signed informed consent. However, patients who met the following criteria were excluded: (1) aged < 18 years old; (2) history of allergy to tigecycline or tetracycline; (3) inability to collect blood samples for pharmacokinetic analysis; and (4) life expectancy less than 1 month due to end-stage diseases and unlikely to complete the course of treatment. Due to the observational nature, the dose of tigecycline was decided by the chief physician. Other combined therapies were not intervened either. A consecutive total sampling strategy was employed in this study, thus, all potential eligible patients in our ICU were screened during the study period and enrolled if eligible.

### 2.3 Clinical follow-up

The primary outcome was clinical response, which was defined as the disappearance or improvement in signs and biochemical indicators of infection (e.g., more than 50% reduction in C-reactive protein or procalcitonin) and was evaluated 1–2 days after the end of tigecycline treatment. The secondary outcomes were 28-mortality rate and major adverse events, mainly hepatic functions, prothrombin time (PT) and serum fibrinogen level (FG). The outcomes were assessed by two researchers independently, but no blinding was performed. The patients were closely monitored, and the following data were collected: (1) patient characteristics; (2) microbiological and laboratory results which were reported by the clinical lab; and (3) regimen of tigecycline and the use of other anti-microorganism drugs.

### 2.4 Blood sample collection and PK analysis

Blood samples for pharmacokinetic analysis were collected immediately before the seventh drug administration and at 0.5, 1, 2, 3, 4, 6, and 12 h postdose from the central vein. The blood samples were centrifuged at 3000 r/min for 10 min at 4 °C, and then the plasma samples were isolated and stored at -80 °C before determination. The tigecycline concentrations were determined by a validated LC–MS/MS method (Shao et al., 2018). The PK parameters of each patient were calculated based on two previously developed population PK models (Song et al., 2024; Su et al., 2024), and 24-hour AUC at steady status was the main parameter of interest.

### 2.5 Statistical analysis

The sample size referred to conventional PK/PD research and was not estimated. All continuous data are represented as the mean ± standard deviation or median and interquantile ranges. Categorical data are represented as numbers and percentages. Records containing missing values were omitted from the statistical analysis. To identify any factor (especially tigecycline exposure) associated with clinical outcomes, patients were divided into two groups based on outcomes, and then univariate logistic regression analysis was performed. Factors with P<0.05 in univariate regression were put into stepwise backward multivariate logistic regression analysis. However, correlation analysis was performed *a priori* to assess the correlation between factors, and only one factor of those with r > 0.3 could be put into the multivariate analysis. The selection was based on professional knowledge and the feasibility of obtaining the factor in clinical practice. Finally, factors with P < 0.05 in the multivariate analysis were independent factors associated with clinical outcomes. Subgroup analysis based on patient characteristics was also performed. All statistical analyses were performed in SPSS 26.

## 3 Results

A total of 130 critically ill patients were included in the study, and the demographic characteristics are shown in **Table 1**. The most common infection type was lower respiratory tract infection (LRTI), and CRAB was the most common pathogen. The median MIC of pathogens was 1 mg/mL for both CRPK and CRAB. Tigecycline monotherapy was only 13.8%, and the frequently used combination was cefoperazone-sulbactam. Most patients (123/130) received tigecycline at a maintenance dose of 50 mg every 12 hours, and the other 7 patients received a maintenance dose of 100 mg every 12 hours.

**Table 1:** The demographic characteristic of included patients and subgroups.

Variable	Total (n=130)	Subgroups-1		Subgroups-2	
		Response (n=65)	Failure (n=65)	Survival (n=83)	No survival (n=47)
Sex, n (%)					
Male	91(70.0%)	47(72.3%)	44(67.7%)	61(73.5%)	30(63.8%)
Female	39(30.0%)	18(27.7%)	21(32.3%)	22(26.5%)	17(36.2%)
Age (years)	68.0(56.0,77.8)	66.0(53.0,78.0)	68.0(58.0,77.0)	66.0(53.0,75.5)	70.0(61.5,79.5)
Height(cm)	166(160,172)	168(160,172)	165(158,170)	168(160,172)	165(157,170)
Weight (kg)	62.0(55.0,75.0)	65.0(55.0,75.0)	60.0(54.8,70.0)	65.0(55.5,75.0)	58.0(52.6,68.8)
BMI (kg/m^2^)	23.4(20.8,25.5)	23.6(20.8,25.7)	22.9(20.3,24.7)	24.1(21.3,25.7)	21.8(20.0,24.3)
Initial Empirical Therapy	48(36.9%)	21(32.3%)	27(41.5%)	26(31.3%)	22(46.8%)
Comorbidities, n (%)					
Hypertension	58(44.6%)	25(38.5%)	33(50.8%)	30(36.1%)	28(59.6%)
Atrial Fibrillation	7(5.4%)	3(4.6%)	4(6.2%)	4(4.8%)	3(6.4%)
CHD	16(12.3%)	9(13.8%)	7(10.8%)	12(14.5%)	4(8.5%)
Chronic Pulmonary Disease	17(13.1%)	8(12.3%)	9(13.9%)	9(10.8%)	8(17.0%)
Chronic liver failure	4(3.1%)	2(3.1%)	2(3.1%)	3(3.6%)	1(2.1%)
DM	36(27.7%)	15(23.1%)	21(32.3%)	21(25.3%)	15(31.9%)
Chronic renal failure	12(9.2%)	5(7.7%)	7(10.8%)	7(8.4%)	5(10.6%)
Immunosuppression drug	20(15.4%)	5(7.7%)	15(23.1%)	7(8.4%)	13(27.7%)
Malignancy	42(32.3%)	19(29.2%)	23(35.4%)	20(24.1%)	22(46.8%)
Past medical history and personal history, n (%)					
Smoking history	8(6.2%)	4(6.2%)	4(6.2%)	6(7.2%)	2(4.3%)
Alcohol history	13(10.0%)	8(12.3%)	5(7.7%)	11(13.3%)	2(4.3%)
Surgical history	68(52.3%)	29(44.6%)	39(60.0%)	42(50.6%)	26(55.3%)
Trauma history	8(6.2%)	6(9.2%)	2(3.1%)	7(8.4%)	1(2.1%)
APACHE II score	27(21,32)	25(20,29)	29(22,33)	25(20,31)	29(24,33)
SOFA score	7(4,10)	6(3,8)	8(5,11)	6(4,8)	9(7,12)
Infected site, n (%)					
IAIs	49(37.7%)	28(43.1%)	21(32.3%)	34(41.0%)	15(31.9%)
LRTIs	76(58.5%)	34(52.3%)	42(64.6%)	49(59.0%)	27(57.4%)
BSIs	37(28.5%)	19(29.2%)	18(27.7%)	18(21.7%)	19(40.4%)
Other or undefined	26(20.0%)	13(20.0%)	13(20.0%)	16(19.3%)	10(21.3%)
Pathogen, n (%)					
CRKP	48(36.9%)	23(35.4%)	25(38.5%)	29(34.9%)	19(40.4%)
MIC	1(0.5,2)	1(0.625,1)	1(0.5,2)	1(1,2)	1(0.5,1)
CRAB	64(49.2%)	32(49.2%)	32(49.2%)	40(48.2%)	24(51.1%)
MIC	1(1,2)	1(1,1)	1(1,2)	1(1,2)	1(1,1.5)
Others	62(47.7%)	30(46.2%)	32(49.2%)	39(47.0%)	23(48.9%)
Mechanical ventilation	95(73.1%)	44(67.7%)	51(78.5%)	59(71.1%)	36(76.6%)
CRRT	26(20.0%)	11(16.9%)	15(23.1%)	14(16.9%)	12(25.5%)
ECMO	3(2.3%)	1(1.5%)	2(3.1%)	1(1.2%)	2(4.3%)
Mixed bacterial infection	55(42.3%)	25(38.5%)	30(46.2%)	31(37.3%)	24(51.1%)
Mixed Fungi infection	20(15.4%)	6(9.2%)	14(21.5%)	9(10.8%)	11(23.4%)
Combined antibiotics, n (%)					
Tigecycline monotherapy	18(13.8%)	8(12.3%)	10(15.4%)	13(15.7%)	5(10.6%)
Polymyxins	25(19.2%)	8(12.3%)	17(26.2%)	13(15.7%)	12(25.5%)
Cefoperazone-Sulbactam	37(28.5%)	27(41.5%)*	10(15.4%)*	30(36.1%)	7(14.9%)
Ceftazidime-Avibactam	8(6.2%)	5(7.7%)	3(4.6%)	6(7.2%)	2(4.3%)
Piperacillin-Tazobactam	11(8.5%)	3(4.6%)	8(12.3%)	5(6.0%)	6(12.8%)
Carbapenems	26(20.0%)	11(16.9%)	15(23.1%)	12(14.5%)	14(29.8%)
Antifungal drugs	39(30.0%)	17(26.2%)	22(33.8%)	18(21.7%)	21(44.7%)
Others	21(16.2%)	11(16.9%)	10(15.4%)	12(14.5%)	9(19.1%)
C_min_(mg/L)	0.386(0.23,0.597)	0.302(0.19,0.478)	0.469(0.322,0.722)	0.312(0.199,0.519)	0.478(0.36,0.690)
C_max_(mg/L)	1.57(1.00,2.47)	1.37(0.814,1.94)	1.81(1.26,2.91)	1.52(0.844,2.19)	1.77(1.25,3.11)
AUC_24_ (mg·h/L)	14.0(9.48,19.6)	11.2(7.45,16.2)*	16.7(12.8,23.2)*	12.4(7.83,16.9)	17.0(12.3,22.4)
AUCI	14.4(8.03,25.5)	9.90(7.45,16.2)	19.8(9.45,27.5)	10.6(7.45,19.8)	20.9(12.1,27.5)
Laboratory data					
MAP (mmHg)	62(54,72)	63(54,78)	59(54,68)	63(54,79)	59(54,65)
HR (bpm)	115(91,133)	115(90,133)	114(94,133)	115(88,133)	113(95,133)
R (bpm)	28(23,32)	29(24,34)	27(22,31)	28(24,34)	26(22,32)
PaO_2_ (mmHg)	91(77,109)	94(77,110)	86.3(75,107)	94(78,111)	86(72,102)
AaDO_2_ (mmHg)	170(129,235)	165(130,207)	193(99,290)	162(123,209)	202(145,296)
P/F	224(166,290)	233(185,282)	198(140,300)	235(178,298)	192(140,261)
PH	7.41(7.37,7.47)	7.44(7.39,7.47)	7.39(7.35,7.45)	7.43(7.38,7.47)	7.38(7.34,7.45)
BE (mmol/L)	-0.8(-4.1,3.1)	1.5(-3.4,4.7)	-2.4(-4.2,1.9)	1.1(-3.4,3.9)	-2.9(-4.5,1.6)
Lactate (mmol/L)	1.9(1.3,2.9)	1.6(1.1,2.6)	2.2(1.6,3.2)	1.7(1.2,2.7)	2.3(1.7,3.2)
Cr (μmol/L)	75.5(50,120)	72(50,126)	76(50,116)	72(48.5,126)	84(52,117)
ALT (U/L)	24.5(14,40.8)	21(11,32)	29(17,44)	22(12.5,38)	29(16.5,52)
AST (U/L)	28(19,45.5)	25(18,36)	34(23,50)	26(18,38)	36(22.5,52)
ALP (U/L)	96.5(75.3,132)	99(74,130)	93(76,133)	96(72.5,130)	99(80.5,150)
GGT (U/L)	67.5(32.3,125)	62(31,125)	69(33,112)	72(36,126)	54(30.5,112)
TBIL (μmol/L)	17.2(10.4,30.1)	15.9(9.4,29.3)	18.1(12.3,30.3)	15.9(9.9,29.4)	17.6(12.4,30.4)
DBIL (μmol/L)	9.0(4.3,19.5)	7.1(3.6,16.9)	9.4(5.2,21.0)	7.2(3.6,17.2)	10.8(5.7,20.8)
IBIL (μmol/L)	8.6(5.3,12.9)	7.8(5.4,13.9)	9.0(5.2,12.7)	8.3(5.8,12.9)	8.8(4.4,12.9)
ALB (g/L)	27.4(25.8,30.0)	28.0(26.6,30.8)*	26.5(24.8,28.8)*	27.6(26.1,30.2)	26.5(25.0,29.1)
Hb (g/L)	75.0(66.0,85.0)	77.0(67.0,87.0)	73.0(65.0,82.0)	76.0(66.5,86.5)	73.0(64.5,82.5)
HCT (%)	23.2(20.8,26.3)	23.6(21.3,27.3)	22.7(20.0,25.2)	23.6(21.3,27.3)	22.5(19.7,25.0)
WBC (×10^9^/L)	9.9(6.0,14.0)	9.3(6.4,12.6)	10.6(6.0,14.7)	9.4(6.3,12.9)	10.6(5.5,16.2)
ANC (×10^9^/L)	8.2(4.8,11.8)	7.8(4.9,10.9)	8.5(4.7,13.2)	8.2(4.9,11.0)	8.2(4.6,14.1)
ALC (×10^9^/L)	0.7(0.4,1.0)	0.7(0.4,1.0)	0.6(0.3,1.1)	0.7(0.4,1.0)	0.5(0.3,1.1)
PLT (×10^9^/L)	140(77.0,254)	173(122,309)*	96.0(51.0,184)*	171(121,265)	80.0(44.0,134)
PCT (ng/mL)	0.7(0.3,2.0)	0.5(0.2,1.6)	1.1(0.6,2.9)	0.6(0.2,1.4)	1.4(0.6,4.0)
CRP (mg/L)	135(75.3,197)	110(70.4,174)	158(93.3,202)	115(69.0,176)	165(106,206)
Maximum glucose (mmol/L)	11.1(8.9,13.1)	10.6(8.6,12.5)	11.3(9.7,13.2)	10.6(8.6,12.4)	11.3(10.0,13.5)
Minimum glucose (mmol/L)	7.0(5.7,8.6)	6.9(5.9,8.2)	7.2(5.6,9.0)	7.1(5.9,8.5)	6.8(5.3,8.7)
PT (s)	15.9(14.8,17.4)	15.8(14.8,17.5)	16.0(14.8,17.3)	15.9(14.8,17.4)	16.0(14.7,17.4)
INR	1.3(1.2,1.4)	1.3(1.2,1.5)	1.3(1.2,1.4)	1.3(1.2,1.4)	1.3(1.2,1.4)
APTT (s)	48.7(41.8,57.9)	48.9(40.9,60.7)	48.7(42.1,56.5)	47.3(41.9,57.7)	49.8(42.2,58.6)
FG (g/L)	4.0(3.0,4.9)	4.1(3.0,5.0)	4.0(3.1,4.8)	4.1(3.2,5.0)	3.9(2.8,4.8)
TT (s)	17.0(15.5,18.6)	16.7(15.7,18.4)	17.5(15.3,18.9)	16.7(15.6,18.5)	17.5(15.3,18.9)

*indicates independent risk factors in multivariate logistic analysis. BMI, body mass index; CHD, coronary heart disease; DM, diabetes mellitus; ICU, intensive care unit; APACHE, acute physiology and chronic health evaluation; SOFA, sequential organ failure assessment; IAIs, intra-abdominal infections; LRTIs, lower respiratory tract infections; BSIs, bloodstream infections; CRKP, carbapenem-resistant *Klebsiella pneumoniae*; CRAB, carbapenem-resistant *Acinetobacter baumannii*; MIC, minimum inhibitory concentration; CRRT, continuous renal replacement therapy; ECMO, extracorporeal membrane oxygenation; C_min_, trough plasma concentration; Cmax, peak plasma concentration; AUC_24_, area under the concentration-to-time curve across 24 hours; AUCI, area under the concentration-to-time curve across 24 hours/the minimum inhibitory concentration; MAP, mean arterial pressure; HR, heart rate; R, respiration; PaO_2_, partial pressure of oxygen in arterial blood; AaDO_2_ alveolar - arterial oxygen difference; P/F, PaO_2/_FiO_2_; PH, potential of hydrogen; BE, base excess; Lactate, lactic acid; Cr, creatinine; ALT, alanine aminotransferase; AST, aspartate aminotransferase; ALP, alkaline phosphatase; GGT, gamma-glutamyl transpeptidase; TBIL, total bilirubin; DBIL, direct bilirubin; IBIL, indirect bilirubin; ALB, serum albumin; Hb, hemoglobin; HCT, hematocrit; WBC, white blood cell; ANC, absolute neutrophil count; ALC, absolute lymphocyte count; PLT, platelet; PCT, procalcitonin; CRP, C-reactive protein; PT, prothrombin time; INR, international normalized ratio; APTT, activated partial thromboplastin time; FG, fibrinogen; TT, thrombin time.

The clinical outcomes of tigecycline treatment are shown in **Table 2**. The overall response rate and 28-day mortality rate were 50.0% and 36.2%, respectively. When the patients were stratified by infection site, intraabdominal infection (IAI) patients had the highest success rate, while blood stream infection (BSI) patients had the highest mortality rate. The results were comparable between patients with different pathogens. In terms of adverse events, lower FG was most common, and indicators of drug-induced livery injury (DILI) should be noted.

**Table 2:** The clinical outcome of tigecycline treatment.

Variable	Total (n=130)	Subgroups-1			Subgroups-2	
		IAIs (n=49)	LRTIs (n=76)	BSIs (n=37)	CRKP (n=47)	CRAB (n=64)
Clinical response rate	65(50.0%)	28(57.1%)	34(44.7%)	19(51.4%)	22(46.8%)	32(50.0%)
Mortality rate	47(36.2%)	15(30.6%)	27(35.5%)	18(48.6%)	18(38.3%)	24(37.5%)
Days of hospital stay	49(31,81)	67(35,99)	49(31,71)	45(30,75)	58(31,83)	50(31,71)
Days of ICU stay	29(14,48)	21(11,50)	37(19,50)	27(14,48)	28(13,47)	38(20,52)
Days of tigecycline treatment	12(8,19)	12(9,20)	12(8,18)	14(9,18)	9(13,22)	13(9,20)
ADRs						
ALT>3×ULN	7(5.4%)	3(6.1%)	5(6.6%)	0(0.0%)	2(4.3%)	4(6.3%)
AST>3×ULN	8(6.2%)	3(6.1%)	5(6.6%)	1(2.7%)	2(4.3%)	5(7.8%)
ALP>2×ULN	13(10.0%)	7(14.3%)	5(6.6%)	6(16.2%)	7(14.9%)	4(6.3%)
TBIL>2×ULN	25(19.2%)	13(26.5%)	14(18.4%)	8(21.6%)	13(27.7%)	12(18.8%)
PT>ULN+3	32(24.6%)	11(22.4%)	19(25.0%)	10(27.0%)	12(70.2%)	16(25.0%)
FG<LLN	51(39.2%)	17(34.7%)	30(39.5%)	15(40.5%)	17(36.2%)	26(40.6%)

IAIs, intra-abdominal infections; LRTIs, lower respiratory tract infections; BSIs, bloodstream infections; CRKP, carbapenem-resistant *Klebsiella pneumoniae*; CRAB, carbapenem-resistant *Acinetobacter baumannii*; ICU, intensive care unit; ADRs, adverse drug reactions; ALT, alanine aminotransferase; AST, aspartate aminotransferase; ALP, alkaline phosphatase; TBIL, total bilirubin; PT, prothrombin time; FG, fibrinogen; ULN, upper limit of normal range; LLN, lower limit of normal range.

ULNs for ALT, AST and ALP were 50, 40 and 125, respectively. ULN for TBIL was 26 μmol/L. ULN for PT was 13.1 s. LLN for FG was 2 g/L.

The tigecycline concentrations in plasma were determined, 24-hour area-undercurves (AUCs) of patients were calculated, and the distributions of AUC and C_min_ are shown in **Figure 1**.

**Figure 1:**
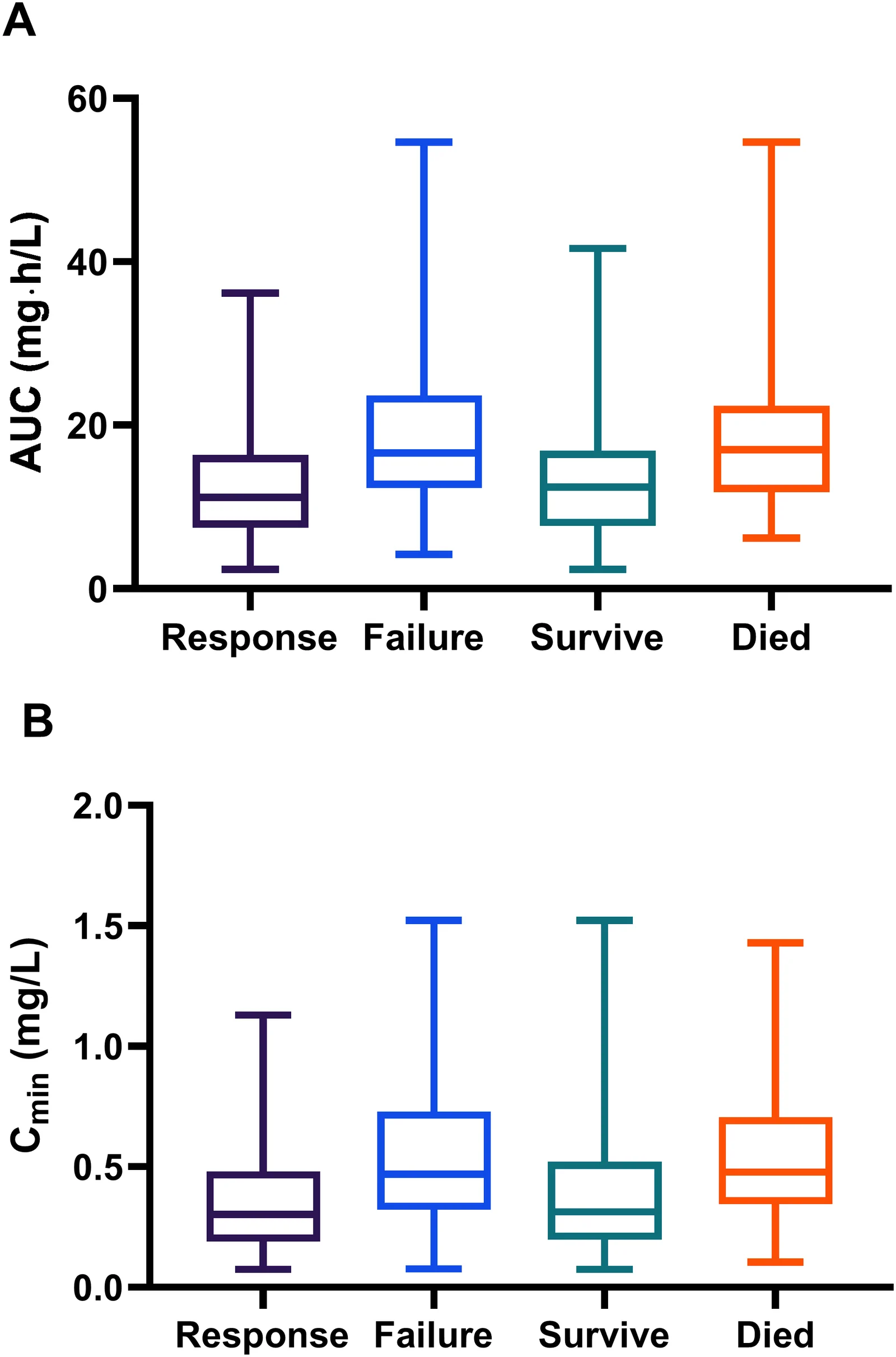
The AUC and C_min_ distributions of tigecycline in patients. **(A)** 24-hour area under the curve of tigecycline; **(B)** C_min_ of tigecycline. Significant differences in AUC and C_min_ exist between patients with different outcomes.

The patients were stratified according to clinical outcome, as shown in **Table 1**. Then, univariate and multivariate analyses were performed to identify independent risk factors (**Table 1**; **Supplementary Tables S1**, **S2**). AUC, albumin level (ALB), platelet count (PLT) and combined use of cefoperazone-sulbactam were independent factors associated with response (**Supplementary Table S1**). Surprisingly, AUC was negatively related to clinical outcome. C_min_ was also an independent risk factor for mortality (**Supplementary Table S2**).

We performed subgroup analysis based on infected pathogens, CRKP and CRAB, and the results are shown in **Supplementary Tables S3**, **S4**. Tigecycline exposure was not a risk factor in these analyses. Risk factors for the most common AE, lower FG, were also analyzed, and the results are shown in **Supplementary Table S5**.

## 4 Discussion

The clinical outcome of tigecycline for critically ill patients with drug-resistant pathogen infection was assessed in this study, and the relationship between clinical outcome and exposure was analyzed. The clinical response rate in various types and pathogen infections was satisfactory, and the risk factors associated (ACU, PLT, ALB and combined use of cefoperazone-sulbactam) with clinical outcome were identified. Higher AUC and C_min_ were unexpectedly associated with poorer clinical response and higher mortality, an observed association likely influenced by disease severity and pharmacokinetic alterations in critically ill patients, which indicated that the PK/PD profile of tigecycline in these patients should be revisited.

This study reported the clinical response rate of tigecycline for drug-resistant pathogen (mainly carbapenem-resistant pathogen) infections. A systemic review and meta-analysis focusing on the clinical outcome of tigecycline-containing regimens for the treatment of MDR-AB showed a pooled clinical response rate of 58.1%, which was similar to our results (Sodeifian et al., 2023). A previous cohort study also found a similar response rate (50-60%) to tigecycline treatment for IAI infection caused by extended-spectrum β-lactamase-producing *Enterobacteriaceae* (Yu et al., 2020). The clinical response rate and mortality rate were also comparable to other choices, such as colistin (Liu et al., 2021). Thus, although recent novel antibiotics showed good efficacy for CRO infection, tigecycline remained one of last choices for these infections.

The clinical outcomes of CRAB- and CRKP-infected patients are similar, which may be due to the high susceptibility of tigecycline to the two pathogens (Wu et al., 2024). Tigecycline had a more favorable clinical response in IAI patients. The intra-abdominal concentration of tigecycline was relatively high, and tigecycline could cover common pathogens of IAI, including gram-negative, gram-positive and anaerobic bacteria (Barbour et al., 2009). In contrast, tigecycline had a poor outcome in BSI patients, and it was suggested against the use of tigecycline due to its low plasma concentration (Niu et al., 2019). Coadministration of cefoperazone-sulbactam was an independent factor of clinical outcome. The sulbactam-containing regime had a synergistic effect with tigecycline against CRAB and was reported to be associated with clinical outcome in CRAB infection (Kubin et al., 2025). The adverse events rate, including lower FG level and abnormal hepatic function indicators, was also similar to previous studies (Yu et al., 2022; Zhu et al., 2024).

The most surprising finding is that tigecycline exposure is negatively related to the clinical outcome. Although several studies have suggested some PK/PD targets for tigecycline, these targets have major shortcomings. Meagher et al. reported an AUC/MIC threshold of 17.9 in cSSTI patients (Meagher et al., 2007). However, this result was only obtained from specific subgroups with gram-positive pathogens, and the exposure-response relationship became uncertain when all pathogens were combined together. Passarell et al. reported an AUC/MIC threshold of 6.96 in IAI patients, but the limitation remained the same (Passarell et al., 2008). A subsequent PK/PD study in CAP only related the PK/PD index to time to defervescence rather than clinical or microbiological response (Rubino et al., 2012). These studies all underscore the importance of generating homogenous populations of pathogens in identifying exposure-response relationships. In our study, the heterogeneity of patient characteristics, including antimicrobial regimens, may also hinder the identification of the PK/PD threshold. However, the treatment of drug resistant pathogens, CRO specifically, requires combination treatment. In multivariate logistic analysis, combined use of cefoperazone-sulbactam was an independent risk factors associated with clinical response, and this may due to the synergistic effect to CRAB (Niu et al., 2019). However, no combined antibiotic option was significant covariate in the analysis for mortality. It should be noted that the negative relationship between exposure and response in this study needs to be correctly interpreted. This finding did not suggest that high exposure to tigecycline is harmful, as the relationship did not indicate causality. Several covariates associated with infection severity are always included in popPK models, and patients with severe infection may have higher exposure (Dai et al., 2025). The tigecycline AUCs were determined during the seventh dose, and the initial exposure was not evaluated. Moreover, the atypical nonlinear plasma-protein-binding behavior may contribute to the result since the unbound fraction of tigecycline decreased with an increase in the total concentration of tigecycline (Mukker et al., 2014). The confounding factors, such as combined antibiotics, would also hinder the interpretation. Besides co-use of cefoperazone-sulbactam, ALB and PLT were independent risk factors in this study, the reason may be that those factors were always associated with severe infection (Wang et al., 2022). It is indicated that special caution should be paid to patients with risk factors in clinical practice.

There are 20% of the included patients received CRRT and 3 patients received ECMO support. The organ support method may influence the tigecycline pharmacokinetic. In our previous study, we have developed population pharmacokinetic model of tigecycline for CRRT patients (Song et al., 2024). When comparing to tigecycline PPK model for patients without CRRT, the parameters were similar, which indicating that tigecycline exposure was not changed under CRRT. The pharmacokinetic of tigecycline in ECMO patient is lack of data (Zhang et al., 2021). The limited sample size for these patients also could not draw a conclusion. However, the effect of ECMO on tigecycline pharmacokinetic would be minimal according to ex vivo study (Zhang et al., 2021). The most common covariates that incorporated in tigecycline population pharmacokinetic models are liver and renal functions (Dai et al., 2025). However, the interindividual variability in tigecycline is large and the exposure in complicated patients, such as patients with septic shock, is difficult to be predicted.

This study also has several limitations. The initial tigecycline exposure was not estimated, which may be important for severe infection. The unbound tigecycline in plasma samples was not determined. A miscellaneous of infected pathogens, infection sites and combined antibiotics may hinder the identification of PK/PD profiles. A larger and more homogenous cohort is needed to identify the exposure-response relationship of tigecycline.

## 5 Conclusion

In this prospective study, the clinical outcome of tigecycline treatment in critically ill patients with drug-resistant pathogens was assessed. The overall response rate and mortality were acceptable, and adverse events were tolerable. ALB, PLT and combined use of cefoperazone-sulbactam were independent factors associated with clinical response. However, in terms of exposure-response analysis, the AUC was negatively associated with clinical response, which calls larger and more homogenous cohorts in future studies.

## Data Availability

The original contributions presented in the study are included in the article/**Supplementary Material**. Further inquiries can be directed to the corresponding authors.
