# Supplementary material for: Clinical outcomes and their relationship with tigecycline exposure in critically ill patients with drug resistant infections
Source: Front Cell Infect Microbiol. 2026 Sep 16;16:1868760. doi: 10.3389/fcimb.2026.1868760 (PMC13623591; doi:10.3389/fcimb.2026.1868760)
Supplement: Supplementary file 1 [file DataSheet1.pdf]

Table S1 Univariate and multivariate logistic analysis for clinical response and failure patients

**Variable**	**Response (n=65)**	**Failure (n=65)**	**Univariate analysis P-value**	**Multivariate analysis OR（95% CI）**	**P-value**
Sex, n (%)			0.566		
Male	47(72.3%)	44(67.7%)			
Female	18(27.7%)	21(32.3%)			
Age (years)	66.0(53.0,78.0)	68.0(58.0,77.0)	0.241		
Height (cm)	168(160,172)	165(158,170)	0.047		
Weight (kg)	65.0(55.0,75.0)	60.0(54.8,70.0)	0.145		
BMI (kg/m^2)	23.6(20.8,25.7)	22.9(20.3,24.7)	0.645		
Initial Empirical Therapy	21(32.3%)	27(41.5%)	0.276		
Comorbidities, n (%)					
Hypertension	25(38.5%)	33(50.8%)	0.159		
Atrial Fibrillation	3(4.6%)	4(6.2%)	0.699		
CHD	9(13.8%)	7(10.8%)	0.594		
Chronic Pulmonary Disease	8(12.3%)	9(13.9%)	0.794		
Chronic liver failure	2(3.1%)	2(3.1%)	1.000		
DM	15(23.1%)	21(32.3%)	0.241		
Chronic renal failure	5(7.7%)	7(10.8%)	0.546		
Immunosuppression drug	5(7.7%)	15(23.1%)	0.020		
Malignancy	19(29.2%)	23(35.4%)	0.454		
immunosuppression drug&Malignancy	20(30.8%)	28(43.1%)	0.147		
Past medical history and personal history					
Smoking history	4(6.2%)	4(6.2%)	1.000		
Alcohol history	8(12.3%)	5(7.7%)	0.384		
Surgical history	29(44.6%)	39(60.0%)	0.080		

Trauma history	6(9.2%)	2(3.1%)	0.164		
Family history	1(1.5%)	3(4.6%)	0.333		
APACHE II score	25(20,29)	29(22,33)	0.046		
SOFA score	6(3,8)	8(5,11)	＜0.001		
Infected site, n (%)					
IAIs	28(43.1%)	21(32.3%)	0.206		
LRTIs	34(52.3%)	42(64.6%)	0.156		
BSIs	19(29.2%)	18(27.7%)	0.846		
Other or undefined	13(20.0%)	13(20.0%)	1.000		
Pathogen, n (%）					
CRKP	23(35.4%)	25(38.5%)	0.716		
CRAB	32(49.2%)	32(49.2%)	1.000		
Others	30(46.2%)	32(49.2%)	0.725		
Mechanical ventilation	44(67.7%)	51(78.5%)	0.169		
CRRT	11(16.9%)	15(23.1%)	0.382		
ECMO	1(1.5%)	2(3.1%)	0.567		
Mixed bacterial infection	25(38.5%)	30(46.2%)	0.375		
Mixed Fungi infection	6(9.2%)	14(21.5%)	0.058		
Concomitant drugs, n (%)					
Tigecycline monotherapy	8(12.3%)	10(15.4%)	0.612		
Polymyxins	8(12.3%)	17(26.2%)	0.050		
Cefoperazone-Sulbactam	27(41.5%)	10(15.4%)	0.001	0.265(0.102-0.693)	0.007
Ceftazidime-Avibactam	5(7.7%)	3(4.6%)	0.470		
Piperacillin-Tazobactam	3(4.6%)	8(12.3%)	0.129		
Carbapenems	11(16.9%)	15(23.1%)	0.382		
Antifungal drugs	17(26.2%)	22(33.8%)	0.340		

Others	11(16.9%)	10(15.4%)	0.812		
C_{min} (mg/L)	0.302(0.19,0.478)	0.469(0.322,0.722)	＜0.001		
AUC_{24} (mg·h/L)	11.2(7.45,16.2)	16.7(12.8,23.2)	＜0.001	1.067(1.015-1.121)	0.011
AUCI	9.90(7.45,16.2)	19.8(9.45,27.5)	0.161		
Laboratory data					
MAP (mmHg)	63(54,78)	59(54,68)	0.123		
HR (bpm)	115(90,133)	114(94,133)	0.939		
R (bpm)	29(24,34)	27(22,31)	0.193		
PaO_2 (mmHg)	94(77,110)	86.3(75,107)	0.838		
$AaDO_2$ (mmHg)	165(130,207)	193(99,290)	0.056		
P/F	233(185,282)	198(140,300)	0.836		
PH	7.44(7.39,7.47)	7.39(7.35,7.45)	0.007		
BE (mmol/L)	1.5(-3.4,4.7)	-2.4(-4.2,1.9)	0.044		
Lactate (mmol/L)	1.6(1.1,2.6)	2.2(1.6,3.2)	0.060		
Cr (μmol/L)	72(50,126)	76(50,116)	0.249		
ALT (U/L)	21(11,32)	29(17,44)	0.358		
AST (U/L)	25(18,36)	34(23,50)	0.199		
ALP (U/L)	99(74,130)	93(76,133)	0.347		
GGT(U/L)	62(31,125)	69(33,112)	0.844		
TBIL (μmol/L)	15.9(9.4,29.3)	18.1(12.3,30.3)	0.079		
DBIL (μmol/L)	7.1(3.6,16.9)	9.4(5.2,21.0)	0.055		
IBIL (μmol/L)	7.8(5.4,13.9)	9.0(5.2,12.7)	0.240		
ALB (g/L)	28.0(26.6,30.8)	26.5(24.8,28.8)	0.002	0.83(0.729-0.945)	0.005
Hb (g/L)	77.0(67.0,87.0)	73.0(65.0,82.0)	0.758		
HCT (%)	23.6(21.3,27.3)	22.7(20.0,25.2)	0.256		
WBC ($\times 10^9$/L)	9.3(6.4,12.6)	10.6(6.0,14.7)	0.260		

ANC ($\times 10^9$/L)	7.8(4.9,10.9)	8.5(4.7,13.2)	0.281		
ALC ($\times 10^9$/L)	0.7(0.4,1.0)	0.6(0.3,1.1)	0.400		
PLT ($\times 10^9$/L)	173(122,309)	96.0(51.0,184)	＜0.001	0.995(0.992-0.999)	0.012
PCT (ng/mL)	0.5(0.2,1.6)	1.1(0.6,2.9)	0.419		
CRP (mg/L)	110(70.4,174)	158(93.3,202)	0.138		
Maximum glucose (mmol/L)	10.6(8.6,12.5)	11.3(9.7,13.2)	0.189		
Minimum glucose (mmol/L)	6.9(5.9,8.2)	7.2(5.6,9.0)	0.507		
PT (s)	15.8(14.8,17.5)	16.0(14.8,17.3)	0.715		
INR	1.3(1.2,1.5)	1.3(1.2,1.4)	0.466		
APTT (s)	48.9(40.9,60.7)	48.7(42.1,56.5)	0.539		
FG (g/L)	4.1(3.0,5.0)	4.0(3.1,4.8)	0.705		
TT (s)	16.7(15.7,18.4)	17.5(15.3,18.9)	0.516		

OR, odds ratio; BMI, body mass index; CHD, coronary heart disease; DM, diabetes mellitus; APACHE, acute physiology and chronic; SOFA, sequential organ failure assessment; IAIs, intra-abdominal infections; LRTIs, lower respiratory tract infections; BSIs, bloodstream infections; CRKP, carbapenem-resistant *Klebsiella pneumoniae*; CRAB, carbapenem-resistant *Acinetobacter baumannii*; CRRT, continuous renal replacement therapy; ECMO, extracorporeal membrane oxygenation; C_{min}, trough plasma concentration; AUC24, area under the concentration-to-time curve across 24 hours; AUCI, area under the concentration-to-time curve across 24 hours/the minimum inhibitory concentration; MAP, mean arterial pressure; HR, heart rate; R, respiration; PaO2, partial pressure of oxygen in arterial blood; AaDO2, alveolar - arterial oxygen difference; P/F, PaO2/FiO2; PH, potential of hydrogen; BE, base excess; Lactate, lactic acid; Cr, creatinine; ALT, alanine aminotransferase; AST, aspartate aminotransferase; ALP, alkaline phosphatase; GGT, gamma-glutamyl transpeptidase; TBIL, total bilirubin; DBIL, direct bilirubin; IBIL, indirect bilirubin; ALB, serum albumin; Hb, hemoglobin; HCT, hematocrit; WBC, white blood cell; ANC, absolute neutrophil count; ALC, absolute lymphocyte count; PLT, platelet; PCT, procalcitonin; CRP, C-reactive protein; PT, prothrombin time; INR, international normalized ratio; APTT, activated partial thromboplastin time; FG, fibrinogen; TT, thrombin time.

Table S2 Univariate and multivariate logistic analysis for 28-day mortality

Variable	Survival (n=83)	No survival (n=47)	Univariate analysis P-value	Multivariate analysis OR（95%CI）	Multivariate analysis P-value
Sex, n (%)					
Male	61(73.5%)	30(63.8%)	0.250		
Female	22(26.5%)	17(36.2%)			
Age (years)	66.0(53.0,75.5)	70.0(61.5,79.5)	0.046		
Height（cm）	168(160,172)	165(157,170)	0.080		
Weight (kg)	65.0(55.5,75.0)	58.0(52.6,68.8)	0.060		
BMI (kg/m2)	24.1(21.3,25.7)	21.8(20.0,24.3)	0.196		
Initial Empirical Therapy	26(31.3%)	22(46.8%)	0.081		
Comorbidities, n (%)					
Hypertension	30(36.1%)	28(59.6%)	0.011	2.744(1.193-6.312)	0.018
Atrial Fibrillation	4(4.8%)	3(6.4%)	0.705		
CHD	12(14.5%)	4(8.5%)	0.327		
Chronic Pulmonary Disease	9(10.8%)	8(17.0%)	0.319		
Chronic liver failure	3(3.6%)	1(2.1%)	0.641		
DM	21(25.3%)	15(31.9%)	0.419		
Chronic renal failure	7(8.4%)	5(10.6%)	0.677		
Immunosuppression drug	7(8.4%)	13(27.7%)	0.005	7.141(2.229-22.883)	0.001
Malignancy	20(24.1%)	22(46.8%)	0.009		
Past medical history and personal history					
Smoking history	6(7.2%)	2(4.3%)	0.503		
Alcohol history	11(13.3%)	2(4.3%)	0.119		
Surgical history	42(50.6%)	26(55.3%)	0.605		
Trauma history	7(8.4%)	1(2.1%)	0.183		

Family history	1(1.2%)	3(6.4%)	0.141		
APACHE II score	25(20,31)	29(24,33)	0.044		
SOFA score	6(4,8)	9(7,12)	＜0.001		
Infected site, n (%)					
IAIs	34(41.0%)	15(31.9%)	0.308		
LRTIs	49(59.0%)	27(57.4%)	0.860		
BSIs	18(21.7%)	19(40.4%)	0.025	0.379(0.154-0.929)	0.034
Other or undefined	16(19.3%)	10(21.3%)	0.784		
Pathogen, n (%）					
CRKP	29(34.9%)	19(40.4%)	0.534		
CRAB	40(48.2%)	24(51.1%)	0.753		
Others	39(47.0%)	23(48.9%)	0.831		
Mechanical ventilation	59(71.1%)	36(76.6%)	0.497		
CRRT	14(16.9%)	12(25.5%)	0.238		
ECMO	1(1.2%)	2(4.3%)	0.296		
Mixed bacterial infection	31(37.3%)	24(51.1%)	0.130		
Mixed Fungi infection	9(10.8%)	11(23.4%)	0.062		
Concomitant drugs, n (%)					
Tigecycline monotherapy	13(15.7%)	5(10.6%)	0.428		
Polymyxins	13(15.7%)	12(25.5%)	0.174		
Cefoperazone-Sulbactam	30(36.1%)	7(14.9%)	0.012		
Ceftazidime-Avibactam	6(7.2%)	2(4.3%)	0.503		
Piperacillin-Tazobactam	5(6.0%)	6(12.8%)	0.194		
Carbapenems	12(14.5%)	14(29.8%)	0.039		
Antifungal drugs	18(21.7%)	21(44.7%)	0.007		
Others	12(14.5%)	9(19.1%)	0.486		

C_{min} (mg/L)	0.312(0.199,0.519)	0.478(0.36,0.690)	0.013	6.261(1.471-26.655)	0.013
AUC_{24} (mg·h/L)	12.4(7.83,16.9)	17.0(12.3,22.4)	0.020		
AUCI	10.6(7.45,19.8)	20.9(12.1,27.5)	0.430		
Laboratory data					
MAP (mmHg)	63(54,79)	59(54,65)	0.073		
HR (bpm)	115(88,133)	113(95,133)	0.953		
R (bpm)	28(24,34)	26(22,32)	0.315		
PaO_2 (mmHg)	94(78,111)	86(72,102)	0.200		
$AaDO_2$ (mmHg)	162(123,209)	202(145,296)	0.025		
P/F	235(178,298)	192(140,261)	0.221		
PH	7.43(7.38,7.47)	7.38(7.34,7.45)	0.025	0(0-0.192)	0.014
BE (mmol/L)	1.1(-3.4,3.9)	-2.9(-4.5,1.6)	0.028		
Lactate (mmol/L)	1.7(1.2,2.7)	2.3(1.7,3.2)	0.294		
Cr (μmol/L)	72(48.5,126)	84(52,117)	0.727		
ALT (U/L)	22(12.5,38)	29(16.5,52)	0.271		
AST (U/L)	26(18,38)	36(22.5,52)	0.116		
ALP (U/L)	96(72.5,130)	99(80.5,150)	0.204		
GGT (U/L)	72(36,126)	54(30.5,112)	0.973		
TBIL (μmol/L)	15.9(9.9,29.4)	17.6(12.4,30.4)	0.185		
DBIL (μmol/L)	7.2(3.6,17.2)	10.8(5.7,20.8)	0.154		
IBIL (μmol/L)	8.3(5.8,12.9)	8.8(4.4,12.9)	0.356		
ALB (g/L)	27.6(26.1,30.2)	26.5(25.0,29.1)	0.080		
Hb (g/L)	76.0(66.5,86.5)	73.0(64.5,82.5)	0.660		
HCT (%)	23.6(21.3,27.3)	22.5(19.7,25.0)	0.172		
WBC ($\times10^9$/L)	9.4(6.3,12.9)	10.6(5.5,16.2)	0.099		
ANC ($\times10^9$/L)	8.2(4.9,11.0)	8.2(4.6,14.1)	0.092		

ALC ($\times 10^9$/L)	0.7(0.4,1.0)	0.5(0.3,1.1)	0.794
PLT ($\times 10^9$/L)	171(121,265)	80.0(44.0,134)	＜0.001
PCT (ng/mL)	0.6(0.2,1.4)	1.4(0.6,4.0)	0.153
CRP (mg/L)	115(69.0,176)	165(106,206)	0.042
Maximum glucose (mmol/L)	10.6(8.6,12.4)	11.3(10.0,13.5)	0.209
Minimum glucose (mmol/L)	7.1(5.9,8.5)	6.8(5.3,8.7)	0.688
PT (s)	15.9(14.8,17.4)	16.0(14.7,17.4)	0.753
INR	1.3(1.2,1.4)	1.3(1.2,1.4)	0.355
APTT (s)	47.3(41.9,57.7)	49.8(42.2,58.6)	0.209
FG (g/L)	4.1(3.2,5.0)	3.9(2.8,4.8)	0.229
TT (s)	16.7(15.6,18.5)	17.5(15.3,18.9)	0.640

OR, odds ratio; BMI, body mass index; CHD, coronary heart disease; DM, diabetes mellitus; APACHE, acute physiology and chronic; SOFA, sequential organ failure assessment; IAIs, intra-abdominal infections; LRTIs, lower respiratory tract infections; BSIs, bloodstream infections; CRKP, carbapenem-resistant *Klebsiella pneumoniae*; CRAB, carbapenem-resistant *Acinetobacter baumannii*; CRRT, continuous renal replacement therapy; ECMO, extracorporeal membrane oxygenation; Cmin, trough plasma concentration; AUC_{24}, area under the concentration-to-time curve across 24 hours; AUCI, area under the concentration-to-time curve across 24 hours/the minimum inhibitory concentration; MAP, mean arterial pressure; HR, heart rate; R, respiration; PaO_2, partial pressure of oxygen in arterial blood; $AaDO_2$, alveolar - arterial oxygen difference; P/F, PaO_2/FiO_2; PH, potential of hydrogen; BE, base excess; Lactate, lactic acid; Cr, creatinine; ALT, alanine aminotransferase; AST, aspartate aminotransferase; ALP, alkaline phosphatase; GGT, gamma-glutamyl transpeptidase; TBIL, total bilirubin; DBIL, direct bilirubin; IBIL, indirect bilirubin; ALB, serum albumin; Hb, hemoglobin; HCT, hematocrit; WBC, white blood cell; ANC, absolute neutrophil count; ALC, absolute lymphocyte count; PLT, platelet; PCT, procalcitonin; CRP, C-reactive protein;PT, prothrombin time; INR, international normalized ratio; APTT, activated partial thromboplastin time; FG, fibrinogen; TT, thrombin time.

Table S3 Univariate and multivariate logistic analysis for clinical response in patients with CRKP infection

**Variable**	**Response (n=23)**	**Failure (n=25)**	Univariate analysis **P-value**	Multivariate analysis	
				**OR（95% CI）**	**P-value**
Sex, n (%)					
Male	18(78.3%)	18(72.0%)	0.618		
Female	5(21.7%)	7(28.0%)			
Age (years)	66.0(47.5,73.5)	70.0(62.0,81.0)	0.186		
Height (cm)	170(165,172)	165(160,172)	0.065		
Weight (kg)	68.0(60.0,77.5)	55.8(51.0,70.0)	0.015		
BMI (kg/m^2)	24.2(22.3,26.1)	22.9(20.2,24.2)	0.027	0.764(0.614,0.952)	0.016
Initial Empirical Therapy	14(60.9%)	8(32.0%)	0.606		
Comorbidities, n (%)					
Hypertension	13(56.5%)	14(56.0%)	0.971		
Atrial Fibrillation	1(4.3%)	3(12.0%)	0.357		
CHD	4(17.4%)	3(12.0%)	0.599		
Chronic Pulmonary Disease	2(8.7%)	2(8.0%)	0.931		
Chronic liver failure	0(0.0%)	1(4.0%)	1.000		
DM	6(26.1%)	12(48.0%)	0.122		
Chronic renal failure	2(8.7%)	4(16.0%)	0.451		
Immunosuppression drug	0(0.0%)	2(8.0%)	0.999		
Malignancy	5(21.7%)	9(36.0%)	0.281		
Past medical history and personal history					
Smoking history	3(13.0%)	0(0.0%)	0.999		
Alcohol history	5(21.7%)	0(0.0%)	0.999		
Surgical history	9(39.1%)	14(56.0%)	0.245		

Trauma history	3(13.0%)	2(8.0%)	0.571		
APACHE II score	26(21,30)	29(22,34)	0.204		
SOFA score	6(3,8)	7(5,10)	0.074		
Infected site, n (%)					
IAIs	16(69.6%)	12(48.0%)	0.134		
LRTIs	7(30.4%)	13(52.0%)	0.134		
BSIs	9(39.1%)	7(28.0%)	0.415		
Other or undefined	6(26.1%)	7(28.0%)	0.882		
Mechanical ventilation	13(56.5%)	18(72.0%)	0.266		
CRRT	8(34.8%)	9(36.0%)	0.930		
Mixed bacteria	10(43.5%)	11(44.0%)	0.971		
Mixed Fungi	1(4.3%)	2(8.0%)	0.607		
Concomitant drugs, n (%)					
Tigecycline monotherapy	2(8.7%)	5(20.0%)	0.280		
Polymyxins	5(21.7%)	7(28.0%)	0.618		
Cefoperazone-Sulbactam	4(17.4%)	1(4.0%)	0.162		
Ceftazidime-Avibactam	5(21.7%)	3(12.0%)	0.372		
Piperacillin-Tazobactam	0(0.0%)	4(16.0%)	0.999		
Carbapenems	6(26.1%)	7(28.0%)	0.882		
Antifungal drugs	7(30.4%)	4(16.0%)	0.241		
Others	4(17.4%)	1(4.0%)	0.162		
MIC (mg/L)	1(0.625,1)	1(0.5,2)	0.523		
C_{max} (mg/L)	1.57(0.781,2.03)	1.89(1.38,2.55)	0.081		
C_{min} (mg/L)	0.307(0.231,0.408)	0.465(0.358,0.722)	0.031		
AUC_{24} (mg·h/L)	11.4(7.80,14.8)	16.4(13.6,22.2)	0.024	1.122(1.021,1.233)	0.016
AUCI	12.5(7.70,17.5)	18.9(12.2,27.6)	0.330		

Laboratory data			
MAP (mmHg)	67(55,77)	60(51,65)	0.385
HR (bpm)	117(91,134)	114(100,135)	1.000
R (bpm)	28(22,34)	26(21,30)	0.623
PaO_2 (mmHg)	99(85,115)	84(75,106)	0.158
$AaDO_2$ (mmHg)	154(132,193)	185(96,245)	0.309
P/F	242(210,283)	223(154,306)	0.579
PH	7.43(7.38,7.46)	7.39(7.34,7.45)	0.339
BE (mmol/L)	-2.1(-5,2.6)	-3.1(-4.4,0.2)	0.520
Lactate (mmol/L)	1.6(1.05,2.6)	2.1(1.6,3)	0.269
Cr (μmol/L)	92(52,170)	88(68,147)	0.465
ALT (U/L)	14(9,28)	23(16,32)	0.482
AST (U/L)	23(15,34.5)	25(15,41)	0.411
ALP (U/L)	99(69,129)	89(66,115)	0.911
GGT (U/L)	61(28.8,136)	67(39,100)	0.678
TBIL (μmol/L)	18(9.5,31.6)	16.8(12.3,29.5)	0.632
DBIL (μmol/L)	10.1(4.45,21)	9.4(5.9,21)	0.482
IBIL (μmol/L)	7.6(5.25,15.7)	8.5(4.8,11)	0.780
ALB (g/L)	27.8(25.9,31.6)	26.9(26.1,28.3)	0.040
Hb (g/L)	74(66.5,80)	72(65,77)	0.430
HCT (%)	23.4(21.6,26.2)	22.5(20.2,23.6)	0.242
WBC ($\times 10^9$/L)	10.3(6.6,14.1)	10.6(6.1,13.7)	0.978
ANC ($\times 10^9$/L)	8.47(4.84,11.0)	9.28(4.6,11.9)	0.942
ALC ($\times 10^9$/L)	0.72(0.585,1.05)	0.69(0.37,0.88)	0.464
PLT ($\times 10^9$/L)	255(138,335)	108(77,184)	0.003
PCT (ng/mL)	0.47(0.175,3.13)	1.39(0.645,3.95)	0.776

CRP (mg/L)	109(67.1,155)	178(108,223)	0.080
Maximum glucose (mmol/L)	10.1(8.6,12.4)	11.6(10.2,13.2)	0.201
Minimum glucose (mmol/L)	7(5.65,7.7)	7.2(5.5,8.9)	0.676
PT (s)	16.3(15.0,17.9)	16.1(14.9,17.4)	0.508
INR	1.35(1.2,1.48)	1.32(1.16,1.45)	0.470
APTT (s)	50.5(42,60.1)	49.8(44.3,54.7)	0.408
FG (g/L)	4(2.82,5.26)	4.08(3.43,4.74)	0.726
TT (s)	16.7(15.9,17.7)	17.3(15.2,18)	0.578

OR, odds ratio; BMI, body mass index; CHD, coronary heart disease; DM, diabetes mellitus; APACHE, acute physiology and chronic; SOFA, sequential organ failure assessment; IAIs, intra-abdominal infections; LRTIs, lower respiratory tract infections; BSIs, bloodstream infections; CRRT, continuous renal replacement therapy; MIC, minimum inhibitory concentration; C_{max}, peak plasma concentration; C_{min}, trough plasma concentration; AUC_{24}, area under the concentration-to-time curve across 24 hours; AUCI, area under the concentration-to-time curve across 24 hours/the minimum inhibitory concentration; MAP, mean arterial pressure; HR, heart rate; R, respiration; PaO2, partial pressure of oxygen in arterial blood; AaDO2, alveolar - arterial oxygen difference; P/F, PaO2/FiO2; PH, potential of hydrogen; BE, base excess; Lactate, lactic acid; Cr, creatinine; ALT, alanine aminotransferase; AST, aspartate aminotransferase; ALP, alkaline phosphatase; GGT, gamma-glutamyl transpeptidase; TBIL, total bilirubin; DBIL, direct bilirubin; IBIL, indirect bilirubin; ALB, serum albumin; Hb, hemoglobin; HCT, hematocrit; WBC, white blood cell; ANC, absolute neutrophil count; ALC, absolute lymphocyte count; PLT, platelet; PCT, procalcitonin; CRP, C-reactive protein; PT, prothrombin time; INR, international normalized ratio; APTT, activated partial thromboplastin time; FG, fibrinogen; TT, thrombin time.

Table S4 Univariate and multivariate logistic analysis for clinical response in patients with CRAB infection

**Variable**	**Subgroups**		Univariate analysis	Multivariate analysis	
	**Success (n=32)**	**Failure (n=32)**	**P-value**	**OR（95% CI）**	**P-value**
Sex, n (%)			0.565		
Male	23(71.9%)	25(78.1%)			
Female	9(28.1%)	7(21.9%)			
Age (years)	73.5(59.5,80.3)	68(60.3,75)	0.407		
Height（cm）	168(160,172)	165(160,170)	0.716		
Weight (kg)	61(52.1,75)	62(55,68.5)	0.899		
BMI (kg/m^2)	22.7(20.4,25.4)	22.5(20.0,24.4)	0.757		
Initial Empirical Therapy	6(18.8%)	8(25.0%)	0.546		
Comorbidities, n (%)					
Hypertension	12(37.5%)	17(53.1%)	0.211		
Atrial Fibrillation	2(6.3%)	1(3.1%)	0.562		
CHD	5(15.6%)	4(12.5%)	0.720		
Chronic Pulmonary Disease	8(25.0%)	4(12.5%)	0.208		
Chronic liver failure	2(6.3%)	0(0.0%)	0.999		
DM	8(25.0%)	11(34.4%)	0.413		
Chronic renal failure	2(6.3%)	3(9.4%)	0.643		
Immunosuppression drug	4(12.5%)	6(18.8%)	0.494		
Malignancy	10(31.3%)	9(28.1%)	0.784		
immunosuppression drug&Malignancy	11(34.4%)	10(31.3%)	0.790		
Past medical history and personal history					
Smoking history	2(6.3%)	3(9.4%)	0.643		
Alcohol history	3(9.4%)	4(12.5%)	0.690		

Surgical history	19(59.4%)	19(59.4%)	1.000		
Trauma history	4(12.5%)	1(3.1%)	0.195		
APACHE II score	26(21,29)	29(23,32)	0.177		
SOFA score	6(4,7)	8(6,10)	0.017		
Infected site, n (%)					
IAIs	3(9.4%)	10(31.3%)	0.039	6.515(1.234,34.389)	0.027
LRTIs	28(87.5%)	25(78.1%)	0.326		
BSIs	8(25.0%)	9(28.1%)	0.777		
Other or undefined	5(15.6%)	5(15.6%)	1.000		
Mechanical ventilation	26(81.3%)	29(90.6%)	0.289		
CRRT	3(9.4%)	7(21.9%)	0.180		
ECMO	1(3.1%)	1(3.1%)	1.000		
Mixed bacteria	13(40.6%)	19(59.4%)	0.136		
Mixed Fungi	3(9.4%)	10(31.3%)	0.039		
Concomitant drugs, n (%)					
Tigecycline monotherapy	3(9.4%)	5(15.6%)	0.454		
Polymyxins	2(6.3%)	11(34.4%)	0.012	6.941(1.204,39.997)	0.03
Cefoperazone-Sulbactam	23(71.9%)	8(25.0%)	<0.001		
Piperacillin-Tazobactam	2(6.3%)	4(12.5%)	0.400		
Carbapenems	3(9.4%)	5(15.6%)	0.454		
Antifungal drugs	6(18.8%)	11(34.4%)	0.162		
Others	3(9.4%)	4(12.5%)	0.690		
MIC	1(1,1)	1(1,2)	0.836		
C_{min} (mg/L)	0.237(0.184,0.486)	0.477(0.338,0.71)	0.003		
C_{max} (mg/L)	1.33(0.793,1.89)	1.64(1.23,2.86)	0.020		
AUC_{24} (mg·h/L)	10.2(7.42,16.3)	16.5(13.2,24.3)	0.007	1.128(1.026,1.239)	0.012

AUCI	9.24(7.13,14.4)	19.3(8.14,25.8)	0.006		
Laboratory data					
MAP (mmHg)	59(54,72)	59(55,68)	0.971		
HR (bpm)	113(89,125)	113(101,130)	0.655		
R (bpm)	30(26,35)	30(25,32)	0.568		
PaO_2 (mmHg)	88(76,97)	95(78,108)	0.167		
$AaDO_2$ (mmHg)	178(159,227)	168(96,291)	0.637		
P/F	200(176,234)	201(161,296)	0.281		
PH	7.45(7.40,7.48)	7.41(7.38,7.46)	0.096		
BE (mmol/L)	1.85(-1.48,4.7)	0.75(-3.2,3.05)	0.187		
Lactate (mmol/L)	1.6(1.1,2.15)	2.2(1.78,3.23)	0.044		
Cr (µmol/L)	70(48.8,119)	73.5(48.8,112)	0.312		
ALT (U/L)	23(15,45.3)	29(17.8,46.3)	0.498		
AST (U/L)	26(18.8,33.8)	39.5(27.3,53)	0.293		
ALP (U/L)	103(84.3,129)	94.5(70.3,173)	0.250		
GGT (U/L)	80.5(47.3,124)	56.5(26.5,115)	0.717		
TBIL (µmol/L)	16.1(9.33,28.1)	17.6(9.25,26.2)	0.219		
DBIL (µmol/L)	7.75(3.53,15.0)	6.2(4.25,17.4)	0.267		
IBIL (µmol/L)	8(5.28,14.1)	9.6(5.98,12.8)	0.187		
ALB (g/L)	27.8(26.8,30.2)	26.4(25.5,29.9)	0.039	0.748(0.577,0.97)	0.029
Hb (g/L)	76.5(67,84.8)	75.5(64.8,81.3)	0.866		
HCT (%)	23.7(20.9,25.5)	23.2(19.8,25.1)	0.560		
WBC ($\times 10^9$/L)	9.15(7.08,11.2)	10.7(6.43,16.3)	0.124		
ANC ($\times 10^9$/L)	7.63(6.00,9.95)	8.69(5.37,14.5)	0.105		
ALC ($\times 10^9$/L)	0.665(0.37,0.97)	0.68(0.345,1.12)	0.205		
PLT ($\times 10^9$/L)	176(133,255)	109(57,199)	0.152		

PCT (ng/mL)	0.29(0.155,0.695)	0.94(0.578,1.92)	0.137
CRP (mg/L)	103(69.2,174)	147(102,196)	0.115
Maximum glucose (mmol/L)	10.5(9.4,11.7)	12.0(10.3,13.2)	0.130
Minimum glucose (mmol/L)	6.89(6.08,8.4)	7.25(6.1,9.05)	0.564
PT (s)	15.7(14.5,16.9)	15.9(14.4,17.1)	0.676
INR	1.26(1.14,1.40)	1.29(1.13,1.40)	0.791
APTT (s)	45.1(40.0,55.7)	47.7(42.4,56.6)	0.783
FG (g/L)	4.19(3.17,5.18)	3.89(3.15,4.72)	0.285
TT (s)	17.0(15.8,18.5)	17.7(15.7,18.9)	0.936

OR, odds ratio; BMI, body mass index; CHD, coronary heart disease; DM, diabetes mellitus; APACHE, acute physiology and chronic; SOFA, sequential organ failure assessment; IAIs, intra-abdominal infections; LRTIs, lower respiratory tract infections; BSIs, bloodstream infections; CRRT, continuous renal replacement therapy; ECMO, extracorporeal membrane oxygenation; MIC, minimum inhibitory concentration; C_{max}, peak plasma concentration; C_{min}, trough plasma concentration; AUC_{24}, area under the concentration-to-time curve across 24 hours; AUCI, area under the concentration-to-time curve across 24 hours/the minimum inhibitory concentration; MAP, mean arterial pressure; HR, heart rate; R, respiration; PaO2, partial pressure of oxygen in arterial blood; AaDO2, alveolar - arterial oxygen difference; P/F, PaO2/FiO2; PH, potential of hydrogen; BE, base excess; Lactate, lactic acid; Cr, creatinine; ALT, alanine aminotransferase; AST, aspartate aminotransferase; ALP, alkaline phosphatase; GGT, gamma-glutamyl transpeptidase; TBIL, total bilirubin; DBIL, direct bilirubin; IBIL, indirect bilirubin; ALB, serum albumin; Hb, hemoglobin; HCT, hematocrit; WBC, white blood cell; ANC, absolute neutrophil count; ALC, absolute lymphocyte count; PLT, platelet; PCT, procalcitonin; CRP, C-reactive protein; PT, prothrombin time; INR, international normalized ratio; APTT, activated partial thromboplastin time; FG, fibrinogen; TT, thrombin time.

Table S5 Univariate and multivariate logistic analysis for lower FG level in over all patients

**Variable**	**Lower FG**		**Univariate analysis**	**Multivariate analysis**	
	**Occurred (n=77)**	**Not occurred (n=51)**	**P-value**	**OR（95%CI）**	**P-value**
Sex, n (%)			0.463		
Male	56(72.7%)	34(66.7%)			
Female	21(27.3%)	17(33.3%)			
Age (years)	64(53,77)	71(62,79.5)	0.011		
Height (cm)	168(160,172)	166(160,172)	0.48		
Weight (kg)	65(56,75)	60(52.6,70)	0.01		
BMI (kg/m^2)	24.1(21.8,25.6)	22.0(19.8,25.2)	0.007	0.877(0.789-0.975)	0.016
Initial Empirical Therapy	29(37.7%)	19(37.3%)	0.963		
Comorbidities, n (%)					
Hypertension	31(40.3%)	27(52.9%)	0.16		
Atrial Fibrillation	4(5.2%)	3(5.9%)	0.867		
CHD	9(11.7%)	6(11.8%)	0.99		
Chronic Pulmonary Disease	4(5.2%)	11(21.6%)	0.009	0.276(0.079-0.972)	0.045
Chronic liver failure	4(5.2%)	0(0.0%)	0.999		
DM	20(26.0%)	16(31.4%)	0.507		
Chronic renal failure	5(6.5%)	6(11.8%)	0.304		
Immunosuppression drug	13(16.9%)	6(11.8%)	0.427		
Malignancy	18(23.4%)	23(45.1%)	0.011		
Past medical history and personal history					
Smoking history	2(2.6%)	6(11.8%)	0.055		
Alcohol history	6(7.8%)	7(13.7%)	0.282		
Surgical history	36(46.8%)	30(58.8%)	0.182		

Trauma history	3(3.9%)	5(9.8%)	0.191
APACHE II score	27(18,33)	26(22,30)	0.768
SOFA score	7(4,10)	7(4,9)	0.562
Infected site, n (%)			
IAIs	32(41.6%)	17(33.3%)	0.349
LRTIs	44(57.1%)	30(58.8%)	0.85
BSIs	22(28.6%)	15(29.4%)	0.918
Other or undefined	14(18.2%)	12(23.5%)	0.463
Pathogen, n (%)			
CRKP	30(39.0%)	17(33.3%)	0.518
CRAB	37(48.1%)	26(51.0%)	0.746
Others	37(48.1%)	25(49.0%)	0.915
Mechanical ventilation	54(70.1%)	39(76.5%)	0.432
CRRT	15(19.5%)	11(21.6%)	0.774
ECMO	2(2.6%)	0(0.0%)	0.999
Mixed bacteria	33(42.9%)	22(43.1%)	0.975
Mixed Fungi	12(15.6%)	8(15.7%)	0.988
Concomitant drugs, n (%)			
Tigecycline monotherapy	12(15.6%)	6(11.8%)	0.544
Polymyxins	18(23.4%)	7(13.7%)	0.182
Cefoperazone-Sulbactam	17(22.1%)	19(37.3%)	0.064
Ceftazidime-Avibactam	4(5.2%)	4(7.8%)	0.547
Piperacillin-Tazobactam	6(7.8%)	4(7.8%)	0.992
Carbapenems	17(22.1%)	9(17.6%)	0.543
Antifungal drugs	25(32.5%)	13(25.5%)	0.399
Others	14(18.2%)	6(11.8%)	0.331

C_{min} (mg/L)	0.348(0.220,0.517)	0.434(0.255,0.695)	0.106		
C_{max} (mg/L)	1.43(0.897,2.22)	1.57(1.31,2.67)	0.231		
AUC_{24} (mg·h/L)	13.3(8.70,16.9)	16.7(10.3,22.8)	0.089		
AUCI (mg·h/L)	12.4(7.54,22.9)	15.4(9.56,27.1)	0.924		
Laboratory data					
MAP (mmHg)	62(54,92)	61(54,68)	0.009	0.98(0.963-0.998)	0.025
HR (bpm)	114(80,135)	117(101,133)	0.261		
R (bpm)	28(22,34)	28(24,32)	0.849		
PaO2 (mmHg)	91(77,110)	93(77,107)	0.52		
AaDO2 (mmHg)	167(123,245)	169(129,224)	0.224		
P/F	228(166,308)	220(173,284)	0.518		
PH	7.41(7.35,7.46)	7.44(7.38,7.48)	0.078		
BE (mmol/L)	-1.3(-4.1,2.6)	0.8(-3.8,3.9)	0.265		
Lactate (mmol/L)	2(1.3,3)	1.7(1.3,2.7)	0.724		
Cr (μmol/L)	76(50,112)	75(49.5,135)	0.711		
ALT (U/L)	29(16,48)	21(11.5,33.5)	0.371		
AST (U/L)	32(19,47)	25(18.5,42.5)	0.939		
ALP (U/L)	99(83,149)	89(72.5,126)	0.461		
GGT (U/L)	81(39,149)	48(30.5,103)	0.232		
TBIL (μmol/L)	18(10.6,29.1)	16.2(9.45,31)	0.63		
DBIL (μmol/L)	9.4(5,18.3)	8.2(3.8,21.9)	0.511		
IBIL (μmol/L)	9.1(5.9,12.7)	7.3(5.25,13.8)	0.959		
ALB (g/L)	27.9(26.2,30.3)	26.8(25.4,28.7)	0.114		
Hb (g/L)	76(66,83)	75(67,85.5)	0.878		
HCT (%)	23.3(20.7,25.2)	22.9(20.9,27.3)	0.739		
WBC ($\times 10^9$/L)	8.2(5.1,12.6)	10.9(7.6,15.4)	0.195		

ANC ($\times 10^9$/L)	7.47(4.33,10.8)	9.28(6.4,13.4)	0.129
ALC ($\times 10^9$/L)	0.68(0.37,1.07)	0.66(0.345,0.84)	0.878
PLT ($\times 10^9$/L)	138(80,247)	167(74.5,262)	0.73
PCT (ng/mL)	0.81(0.36,1.57)	0.71(0.245,3.11)	0.143
CRP (mg/L)	145(75.4,197)	118(75.6,193)	0.513
Maximum glucose (mmol/L)	11(9.2,13)	11.1(8.75,13.8)	0.616
Minimum glucose (mmol/L)	7(5.8,8.9)	7(5.4,8.3)	0.338
PT (s)	15.9(14.8,17.3)	16.2(14.5,17.7)	0.433
INR	1.28(1.18,1.42)	1.32(1.16,1.45)	0.624
APTT (s)	47.4(41.3,53.5)	50.5(42.5,64.1)	0.045
TT (s)	17.4(15.3,18.5)	16.7(15.6,18.8)	0.257

OR, odds ratio;BMI, body mass index; CHD, coronary heart disease; DM, diabetes mellitus; APACHE, acute physiology and chronic; SOFA, sequential organ failure assessment; IAIs, intra-abdominal infections; LRTIs, lower respiratory tract infections; BSIs, bloodstream infections; CRKP, carbapenem-resistant Klebsiella pneumoniae; CRAB, carbapenem-resistant Acinetobacter baumannii; CRRT, continuous renal replacement therapy; ECMO, extracorporeal membrane oxygenation; Cmin, trough plasma concentration; Cmax, peak plasma concentration; AUC_{24}, area under the concentration-to-time curve across 24 hours; AUCI, area under the concentration-to-time curve across 24 hours/the minimum inhibitory concentration; MAP, mean arterial pressure; HR, heart rate; R, respiration; PaO2, partial pressure of oxygen in arterial blood; AaDO2, alveolar - arterial oxygen difference; P/F, PaO2/FiO2; PH, potential of hydrogen; BE, base excess; Lactate, lactic acid; Cr, creatinine; ALT, alanine aminotransferase; AST, aspartate aminotransferase; ALP, alkaline phosphatase; GGT, gamma-glutamyl transpeptidase; TBIL, total bilirubin; DBIL, direct bilirubin; IBIL, indirect bilirubin; ALB, serum albumin; Hb, hemoglobin; HCT, hematocrit; WBC, white blood cell; ANC, absolute neutrophil count; ALC, absolute lymphocyte count; PLT, platelet; PCT, procalcitonin; CRP, C-reactive protein;PT, prothrombin time; INR, international normalized ratio; APTT, activated partial thromboplastin time; FG, fibrinogen; TT, thrombin time.
